# Three new species of the leafhopper genus *Dayus* Mahmood from China (Hemiptera, Cicadellidae, Typhlocybinae, Empoascini)

**DOI:** 10.3897/zookeys.355.6277

**Published:** 2013-11-25

**Authors:** Xiaofei Yu, Maofa Yang

**Affiliations:** 1 Institute of Entomology, Guizhou University, Guiyang Guizhou, 550025, P. R. China; 2 Guizhou Provincial Key Laboratory for Agricultural Pest Management of the Mountainous Region,; 3 Guiyang Guizhou, 550025, P. R. China

**Keywords:** Auchenorrhyncha, leafhopper, taxonomy, morphology

## Abstract

Three new species of the Oriental empoascine leafhopper genus *Dayus* Mahmood are described from China: *D. bifurcatus* **sp. n.**, *D. trifurcatus* **sp. n.** and *D. serratus* **sp. n.** A key to distinguish all Chinese species of the genus is provided.

## Introduction

The Oriental typhlocybine leafhopper genus *Dayus* was established by Mahmood in 1967 with *Dayus elongatus* Mahmood (Singapore) as its type species. Subsequently, Dworakowska (1971) described *Dayus takagii* Dworakowska (Japan) and transferred *Dayus upoluanus* (Osborn, 1934) (Western Samoa) and *Dayus euryphaessus* (Kirkaldy, 1907) (Fiji) to the geuns, Dworakowska and Viraktamath (1978) added a new species: *Dayus formosus* from China (Taiwan) and Qin and Zhang (2007) added three new species from China: *Dayus lii* Qin & Zhang, *Dayus membranaceus* Qin & Zhang and *Dayus lamellatus* Qin & Zhang.

Here we describe three new species of *Dayus* from China and provide a key for the separation of all known Chinese species. The specimens examined are deposited in the Institute of Entomology, Guizhou University, Guiyang, Guizhou, China (GUGC) and The Natural History Museum, London (BMNH).

## Materials and methods

The methods and terminology follow Zhang (1990) except for the nomenclature of wing, for which we follow Dworakowska (1993). Male specimens were dissected under a MOTIC B1 SMS-168 SERIES microscope. Figures were made using an OLYMPUS CX41 and enhanced using Adobe Illustrator CS4. Pictures were taken with VHX-1000C and dealt with by Adobe Illustrator CS4. The body length is measured from the apex of the head to the apex of the forewing.

## Results

### Genus Dayus

Mahmood

http://species-id.net/wiki/Dayus

Dayus Mahmood, 1967: 39.

#### Type species.

*Dayus elongatus* Mahmood, 1967 by original designation.

#### Diagnosis.

**Figures 1–9.**
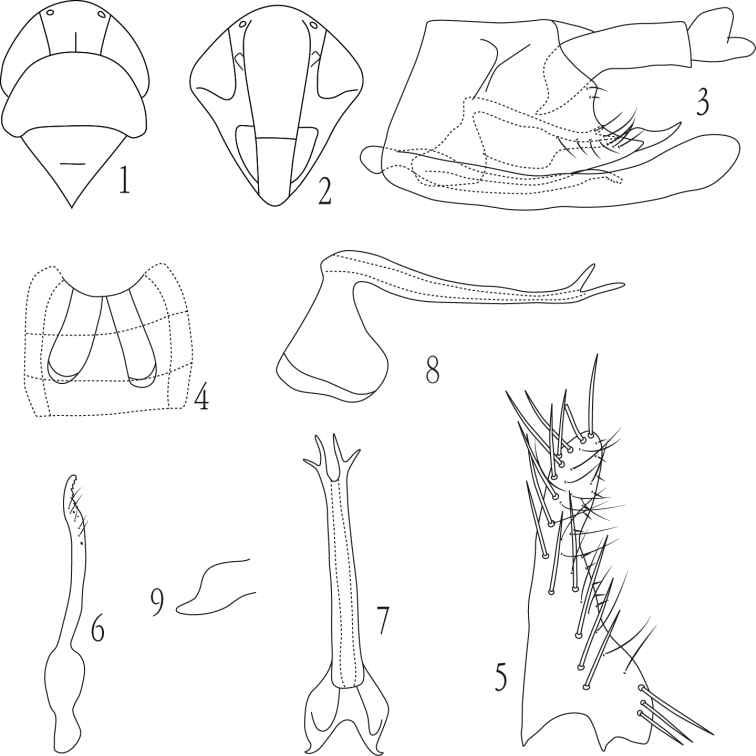
*Dayus bifurcatus* Yu & Yang, sp. n., **1** head and thorax, dorsal view **2** face **3** male genital capsule, lateral view **4** male abdominal apodemes **5** subgenital plate, ventral view **6** paramere **7** aedeagus and connective, dorsal view **8** aedeagus and connective, lateral view **9** anal tube process.

Vertex (Fig. 1) slightly longer medially than next to eye. Forewing (Fig. 22) with 3^rd^ apical cell petiolate, cua cell broad distally; veins RP, MP’ and MP’’+CuA’ arise from m cell. Hindwing (Fig. 23) with apically broad m cell. Male pygofer (Figs 3, 13, 24) abruptly and strongly narrowing caudad; dorsal bridge about half length of pygofer (Figs 15, 26); with few rigid microsetae distally; elongate ventral appendage present, extended beyond pygofer. Subgenital plate (Figs 5, 17, 27) with basal group of macrosetae and one or two oblique rows of more distal macrosetae. Connective (Figs 7, 19, 29) completely fused with the base of aedeagus. Aedeagus (Figs 8, 20, 30) with basal apodeme absent; shaft strongly curved posteriorly at base with one or two pairs of processes.

**Figures 10–21.**
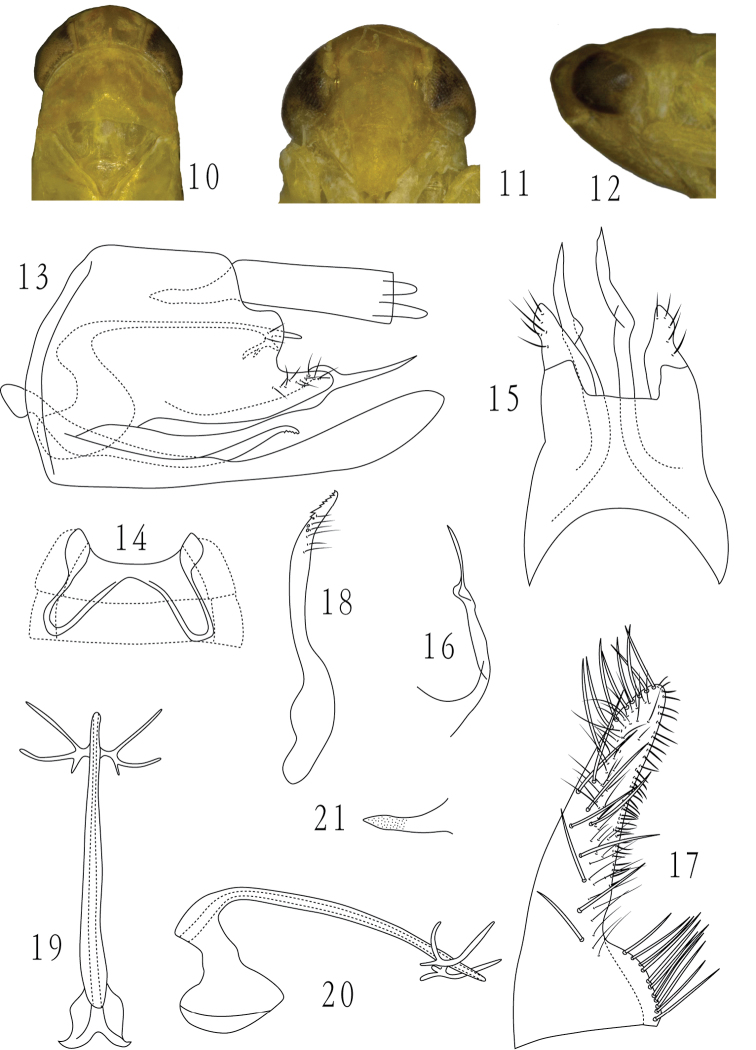
*Dayus trifurcatus* Yu & Yang, sp. n., **10** head and thorax, dorsal view **11** face **12** head and thorax, lateral view **13** male genital capsule, lateral view **14** male abdominal apodemes **15** male pygofer, dorsal view **16** ventral pygofer appendage, outside lateral view **17** subgenital plate, ventral view **18** paramere **19** aedeagus and connective, dorsal view **20** aedeagus and connective, lateral view **21** anal tube process.

**Figures 22–30.**
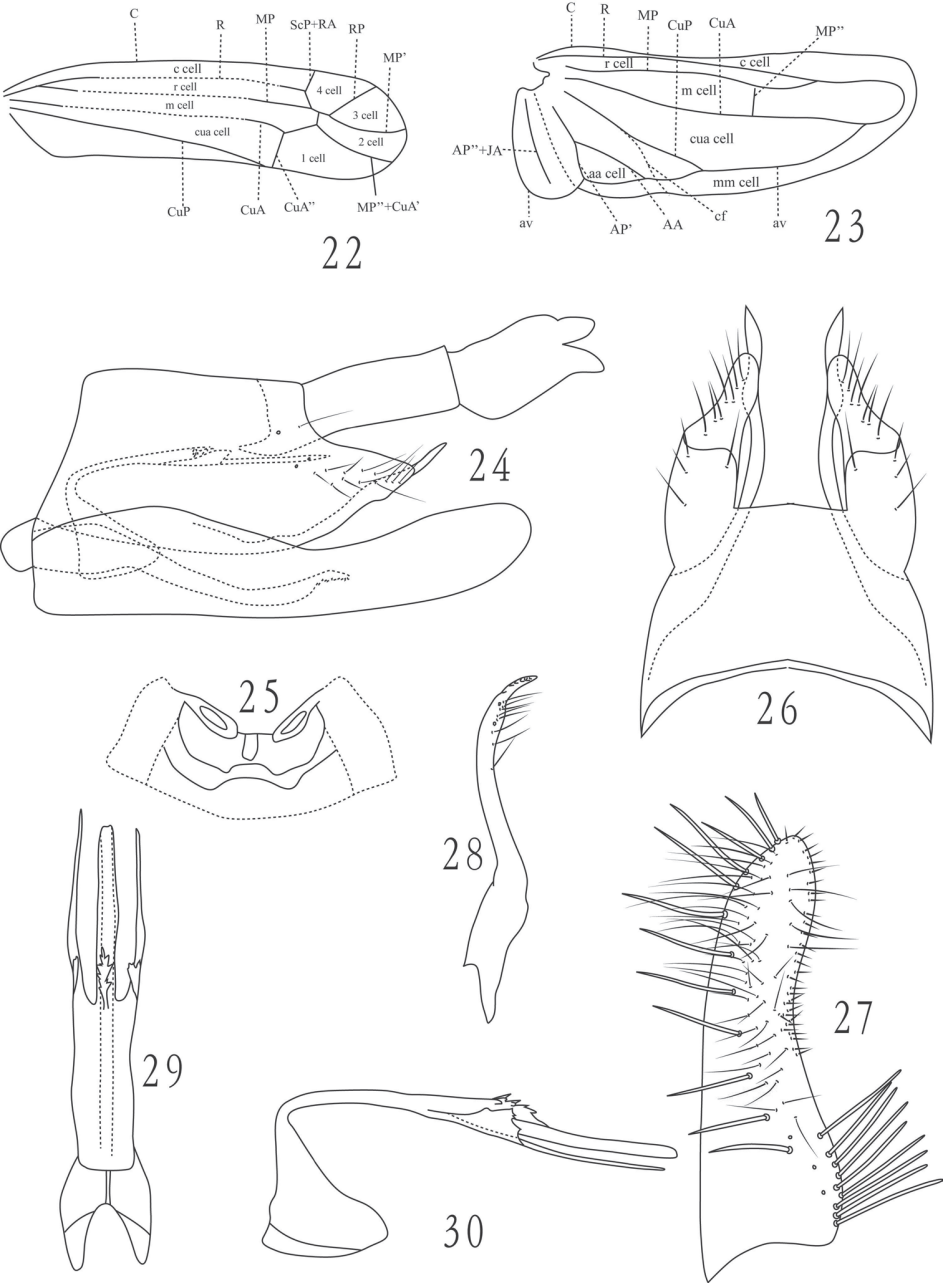
**22–23** *Dayus trifurcatus* Yu & Yang, sp. n., **22** forewing **23** hind wing **24–30** *Dayus serratus* Yu & Yang, sp. n., **24** male genitalia, lateral view **25** male abdominal apodemes **26** male pygofer, dorsal view **27** subgenital plate, ventral view **28** paramere **29** aedeagus and connective, dorsal view **30** aedeagus and connective, lateral view.

#### Key to the Chinese species (males)

**Table:** 

1	Aedeagus with one pair of processes	2
–	Aedeagus with two pairs of processes	6
2	Aedeagal processes with basal serrated lobes (Figs 29–30)	*Dayus serratus* sp. n.
–	Aedeagal processes not dentate basally	3
3	Aedeagal processes unbranched	4
–	Aedeagal processes branched	5
4	Aedeagal processes arising near midlength of shaft	*Dayus membranaceus*
–	Aedeagal processes arising at apex of shaft	*Dayus formosus*
5	Aedeagal processes trifurcate, subapical on shaft (Figs 19–20)	*Dayus trifurcatus* sp. n.
–	Aedeagal processes bifurcate, at apex of shaft (Figs 7–8)	*Dayus bifurcatus* sp. n.
6	Aedeagal processes bifurcate; apical pygofer ventral appendage branched	*Dayus lii*
–	Aedeagal processes not bifurcate; apical pygofer ventral appendage unbranched	7
7	Apical aedeagal processes straight, subapical processes leaf-like	*Dayus lamellatus*
–	Apical aedeagal processes hook-shaped, subapical processes slim	*Dayus takagii*

### Dayus bifurcatus

Yu & Yang sp. n.

http://zoobank.org/8636DDEB-EDC7-4A8C-B6EA-6D7AEF8756DC

http://species-id.net/wiki/Dayus_bifurcatus

Figures 1–9

#### Description.

Length, male 3.0 mm.

General color reddish to reddish brown. Both sides of coronal suture with a brownish patch.

Male ventral abdominal apodemes reaching segment 5 (Fig. 4). Male pygofer with dorsoposterior margin sinuate; pygofer appendage with dorsal margin sinuate in lateral view, tapering to apex (Fig. 3). Subgenital plate about twice as broad basally than distally, with three lateral macrosetae in basal group, an oblique line of 14 macrosetae and several long fine setae subbasally to apex and ca.12 short microsetae at outer margin (Fig. 5). Paramere as in Fig. 6. Aedeagus shaft long and narrow, slightly depressed dorsoventrally, similar in width throughout length in ventral view, with a pair of short bifurcate apical processes (Figs 7, 8). Anal tube process short (Fig. 9).

#### Type material.

Holotype male. China: Zhejiang Province, Fengyang mountain, 30 July 2009, coll. Junqiang Ni.

#### Etymology.

The new species name alludes to the pair of apical bifurcate aedeagal processes.

#### Remarks.

The new species can be distinguished mainly by the shape of the aedeagal shaft and its process configuration as noted in the description.

### Dayus trifurcatus

Yu & Yang sp. n.

http://zoobank.org/1FCC60EB-60FC-490D-8E7B-7E69594D814C

http://species-id.net/wiki/Dayus_trifurcatus

Figures 10 –23

#### Description.

Length, male 4.5–4.6 mm, female 4.7–4.8 mm.

General color yellowish.

Male ventral abdominal apodemes reaching segment 4 (Fig. 14). Male pygofer with dorsoposterior margin strongly sinuate (Fig. 13); ventral appendage expanded at distal 2/3, thereafter abruptly tapering to spine-like apex (Figs 13, 16). Subgential plate abruptly expanded laterobasally about twice as broad basally than distally; with 11 lateral macrosetae in basal group, an oblique line of 17 macrosetae and several long fine setae subbasally to apex and ca.35 short microsetae at outer margin (Fig. 17). Paramere as in Fig. 18. Aedeagus shaft very long and narrow, cylindrical, nearly straight in lateral view, with a subapical trifurcate process on each side, branches slender (Figs 19, 20). Anal tube process relatively long (Fig. 21).

#### Type materials.

Holotype male. China: Beipei, Chongqing, 6 May 2008, coll. Zaihua Yang. Paratypes, 13♂♂, 5♀♀, same data as holotype (GUGC and 1♂, 1♀ in BMNH).

#### Etymology.

The new speciesnamealludes to the trifurcate processes of the aedeagus.

#### Remarks.

Thenew species can be distinguished mainly by the strongly sinuate posterior margin of the pygofer and shape of the aedeagal shaft and configuration of its process as noted in the description.

### Dayus serratus

Yu & Yang sp. n.

http://zoobank.org/A163BC19-73A4-459A-BA07-125F00EF9D8A

http://species-id.net/wiki/Dayus_serratus

Figures 24–30

#### Description.

Length, male 3.9mm.

General color yellowish.

Male ventral abdominal apodemes reaching segment 3 (Fig. 25). Male pygofer (Fig. 24) with dorsoposterior margin concave, tapering caudally; ventral appendage with dorsal margin slightly sinuate, tapered to acute apex. Subgenital plate slightly broader basally, with 9 apically rounded lateral macrosetae in basal group, an oblique line of 12 macrosetae and several long fine setae sub-basally to apex and ca.32 short microsetae at outer margin (Fig. 27). Parameres as in Fig. 28. Aedeagus shaft long, basal half strongly dorsoventrally depressed, distal half narrow and cylindrical, serrate laterally at base on dorsal surface; with two long processes arising at midlength on each side of shaft, basally each process with a lateral lamellate serrate lobe (Figs 29, 30). Anal tube process short (Fig. 24).

#### Type material.

Holotype male. China: Hainan Province, Wuzhi mountain, 13 April 2009, coll. Zaihua Yang.

#### Etymology.

The new species name is derived from the serrationsat midlength of the aedeagal shaft and base of the aedeagal processes.

#### Remarks.

Thenew species can be distinguished mainly by the relatively rather uniform width of the subgenital plate and shape of the aedeagal shaft and configuration of its process as noted in the description.

## Supplementary Material

XML Treatment for Dayus

XML Treatment for Dayus bifurcatus

XML Treatment for Dayus trifurcatus

XML Treatment for Dayus serratus
